# A decade of *GigaScience*: What can be learned from half a million RRIDs in the scientific literature?

**DOI:** 10.1093/gigascience/giac058

**Published:** 2022-06-14

**Authors:** Anita Bandrowski

**Affiliations:** Department of Neurosciences and Center for Research in Biological Systems, University of California, San Diego, La Jolla, California, 92093, United States of America

## Abstract

Research resource identifiers (RRIDs) are persistent unique identifiers for scientific resources used to conduct studies such as reagents and tools. Inclusion of these identifiers into the scientific literature has been demonstrated to improve the reproducibility of papers because resources, like antibodies, are easier to find, making methods easier to reproduce. RRIDs also dramatically reduce the use of problematic resources, such as contaminated cell lines. The addition of RRIDs into a manuscript means that authors have to look up information that they may have previously omitted or confront information about problems that may have been reported about their resources. The use of RRIDs is primarily driven by champion journals, such as *GigaScience* and others. Although still nascent, this practice lays important groundwork for citation types that can cover non-traditional scholarly output, such as software tools and key reagents; giving authors of various types of tools scholarly credit for their contributions.

## Background

RRIDs are not for citing ideas or data since well-developed citation systems already exist for that. Instead, RRID numbers are created by repositories that distribute the research resource, such as Addgene for plasmids, or the National Xenopus Resource for transgenic frogs. RRIDs can also come from registries that govern a resource type, such as the antibodyregistry.org for antibodies or Cellosaurus for cell lines, with the stipulation that RRIDs are lists of resources that can be used as catalogs, as opposed to data about resources (eg., x-ray structures of an antibody, or embryonic ferret images). RRIDs were intended to be used in the methods sections of manuscripts or materials tables, since these were considered to be the least disruptive sections for authors to update.

RRIDs for organisms are largely based on the stock center codes that authors already use to order animals or other resources. Stock centers and other shared facilities are usually supported by grants and need accurate and complete information about the stocks and services they provide for continued grant support. The use of RRID numbers for organisms enables stock centers to track the organisms they distribute a little more easily because journals remind authors to use RRIDs. RRIDs also function as a quick check for authors to ensure that the information in the manuscript matches the information from the stock center.

RRIDs can also be generated by the model organism databases (MODs), which in many cases are the epicenters of the organism community. The MODs tend to focus their efforts on gathering key information about genetics, linking genotypes to phenotypes, and on standardizing  the naming conventions for organism literature, in order to improve our understanding of health and disease. MOD identifiers are omnipresent in the scientific literature, but these are generally not RRIDs for stocks, which has been a source of some confusion, so authors are asked to consult the RRID portal to determine the appropriate identifier for their organism; so, they do not inadvertently identify a transgenic insertion instead of the organism itself. Ideally, MOD-approved organism names, with links to key genomic and phenomic features would be consistently listed by all stock centers as well as all commercial organism suppliers, giving authors of manuscripts the same information across multiple platforms reducing author confusion as they report on their findings.

### RRIDs let readers know which key resources are used in the study

At its surface, the question of which resource was used is so basic that it should take no time to answer; unfortunately in practice, this very simple question is difficult to answer because authors tend to refer to research resources with less than sufficient information [1]. In cases such as salts or buffers, it generally does not matter which precise reagent was used because they are the same. However, with key biological resources, which tend to be more variable and usually the reason that a particular experiment succeeds or fails, the information that authors provide must be highly accurate or they risk making the paper not reproducible without contacting authors, something that Errington and colleagues [2] documented for every single paper they attempted to reproduce in the Cancer Reproducibility Project. Asking authors years after a paper was published to identify a reagent should not be how we report on our science.

Antibodies appear especially vulnerable as companies remove catalog listings when stocks have run out (polyclonals), have been discontinued, or fail some validation test. While many antibody reagents last in deep freezers for decades, the companies that created them may be long gone. Indeed, one of the first antibodies that was ever cited with an RRID in a 2014 paper was a Chemicon antibody, a company that was acquired by Millipore in 2006; but according to google scholar (April 2022), Chemicon continues to be cited in hundreds of recent papers. It should be noted that the name of the company that offered these products has changed at least 6 times through mergers and acquisitions over the last decade. Many of the original Chemicon antibodies are distributed by Merck under the Millipore Sigma brand. In the absence of an extant company with a policy about maintaining sunset records, it is unknowable which antibody was used in a paper.

Antibody problems have long been opprobriated as a major driver of irreproducibility [3] and many solutions have been proposed to alleviate the problems with antibodies ranging from eliminating all polyclonals [4], to adding validation data for each antibody used [5]. In contrast to these somewhat more dramatic proposals, the RRID approach was to simply ask authors to find the RRID numbers and paste them into their manuscript. On the surface, this solution does little for reproducibility, but it does define with much greater certainty which resources were used in a study.

### RRID History

RRIDs came into existence as a three month pilot project started by a group of journal editors, program officers from the National Institutes of Health, and a small number of informaticists. The  first meeting was funded by the International Neuroinformatics Coordinating Facility (incf.org), where 25 journal editors discussed resource-related problems at the Society for Neuroscience conference in New Orleans in 2012. Editors were in disbelief that resources were being cited without sufficient information. The second meeting was a two day workshop at the National Institutes of Health, thanks to the National Institute on Drug Abuse (NIDA), where consensus was reached that, at least, antibodies were problematic and a rough agreement was reached for what should be done (asking authors for RRIDs). In the fall of 2013, during the Society for Neuroscience conference, a smaller group met thanks to Wiley, which established a timeline and goals for the three month pilot project. And in February of 2014, 25 journals started asking authors to provide RRIDs. However, editors did not want to add instructions to 25 different databases, so RRIDs had to be available via a single web portal that authors could be sent to and the process should not put undue stress on the authors. Although there was also no dedicated funding for the project, the National Institute of Diabetes and Digestive and Kidney (NIDDK) information network funded the creation of the SciCrunch portal (https://scicrunch.org/) and data sources, including the antibody and tools registry. The organism stock centers were already available via the Neuroscience Information Framework (NIF). The goal of the pilot project was to see whether authors could put RRIDs into manuscripts and whether the information was correct.

In February of 2014 two journals began asking authors to add RRIDs into their manuscripts. Other journals came on board later after some cajoling, staggering the start time for the 3 month pilot project. The first 100 papers with RRIDs were published within a few months of the pilot starting, and the results showed a dramatic shift in the ability to identify research resources [6]. At that point, journals requesting authors to add RRIDs mostly wanted to continue, and new journals also changed their instructions for authors to include RRIDs. We are currently entering the eighth year of the three month pilot project, and over 1,000 journals have now either added RRIDs to their instructions to authors, publishing checklists, or have mandated their use (https://www.rrids.org/journals). One of the last journals to come on board from the original 25 was *Neuron*, yet the journal [7] spearheaded the most visible place where RRIDs appear  - the STAR (Structured, Transparent, Accessible Reporting) Methods format. The STAR method format was made mandatory across all Cell Press journals, and asks authors to list all of the resources they used in the study, defining where they were obtained, and adding identifiers including RRIDs for each resource.

RRIDs have also expanded beyond the original data scope, initially with the addition of the Cellosaurus database (https://web.expasy.org/cellosaurus/). This came at the request of Cell editors and our NIH (National Institutes of Health) program officer, who knew that problematic cell lines littered the literature, and hoped that one way to combat the issue would be to confront authors with validation information before they published. In 2016, Cellosaurus joined the RRID initiative - adding this important resource to the RRID umbrella. After the first ∼500 papers with cell line RRIDs were published, Babic *et al*. [8] found that incidences of use of problematic cells was 66% lower than the literature without RRIDs. This indicates that RRIDs can reduce the usage of problematic resources. In 2018 Addgene joined at the request of Springer/Nature. Much more recently, the BioSamples database became another authority for RRIDs, this time for biosamples, such as finite cells and tissues including pancreatic islets (BioSamples FAQs 2018; https://www.ebi.ac.uk/biosamples/docs/faq#_why_are_some_biosamples_linked_to_rrids). Unlike the initial sources, the additions to the RRID project included social media campaigns, and both the authority for the resource and the RRID portal updated their websites in close collaboration, asking authors to cite the resources consistently.

The most recent and still nascent efforts to expand the scope of the RRID system are to include core facilities and scientific instruments. The effort was led by the ABRF (Association of Biomolecular Resource Facilities), ABRF Core Marketplace and the libraries of Florida State University, who cataloged and aligned the names of the ABRF core-listed instruments (such as confocal microscopes or sequencers). Demonstrating that the utilization of core facilities is important to their continued support as it is with stock centers. Therefore, many cores have become RRID champions, asking core users to acknowledge their facilities using the RRID via the core website, within instrument scheduling systems such as StratoCore, in protocols, and even in the core leaders email signature.  In the first year, we have already found >400 RRID citations for these Core Facilities, about a third of which are in journals that do not routinely include RRIDs, suggesting that many authors are doing this without being asked by journals.

#### Why did RRIDs become successful?

Champions of RRIDs, including *GigaScience*, are the reason that the project has been successful, without the hard working editors who go after authors, the project would be far behind. Interestingly, the fastidious use of RRIDs has enabled the refinement of at least two machine learning algorithms. The SciScore tool can suggest to authors that a tool, such as SPSS, has a certain RRID (SPSS, RRID:SCR_002865). This is based on a large training dataset of ∼10K sentences that specify the tool with no ambiguity [9]. This training set is largely based on the effort of *GigaScience* and *eLife* staff who have verified these identifiers. The other unexpected result was that Hsu *et al*. [10] were able to automatically discover potential antibody problems if authors use RRIDs in papers. It turns out that even if authors report that some antibody is non-specific, the ability to determine which antibody authors are talking about is generally a difficult task for AI (artificial intelligence). If authors use RRIDs, finding the right antibody in a short list, as opposed to millions, enables the robot to do this previously impossible task.

Currently, the RRID website has been accessed by over a million users (according to google analytics), and there have been three million page views, rising steadily from ∼9K to ∼60K per month (2014 to 2022). Most users (44%) find the portal using search “RRID” and “SciCrunch” as the most common search terms, while referral traffic (21%) comes from eLife, Editorial Manager, Oxford University Press, and Wiley most commonly, suggesting that authors or reachers are coming from the publishers. Direct traffic (33%) rounds out 98% of the total traffic sources to the RRID portal. Some of the referral traffic is coming through the resolver services suggesting that authors are clicking on the linked RRIDs in journals, perhaps to find out more information about the antibody or organism they are interested in.

**Figure 1:**
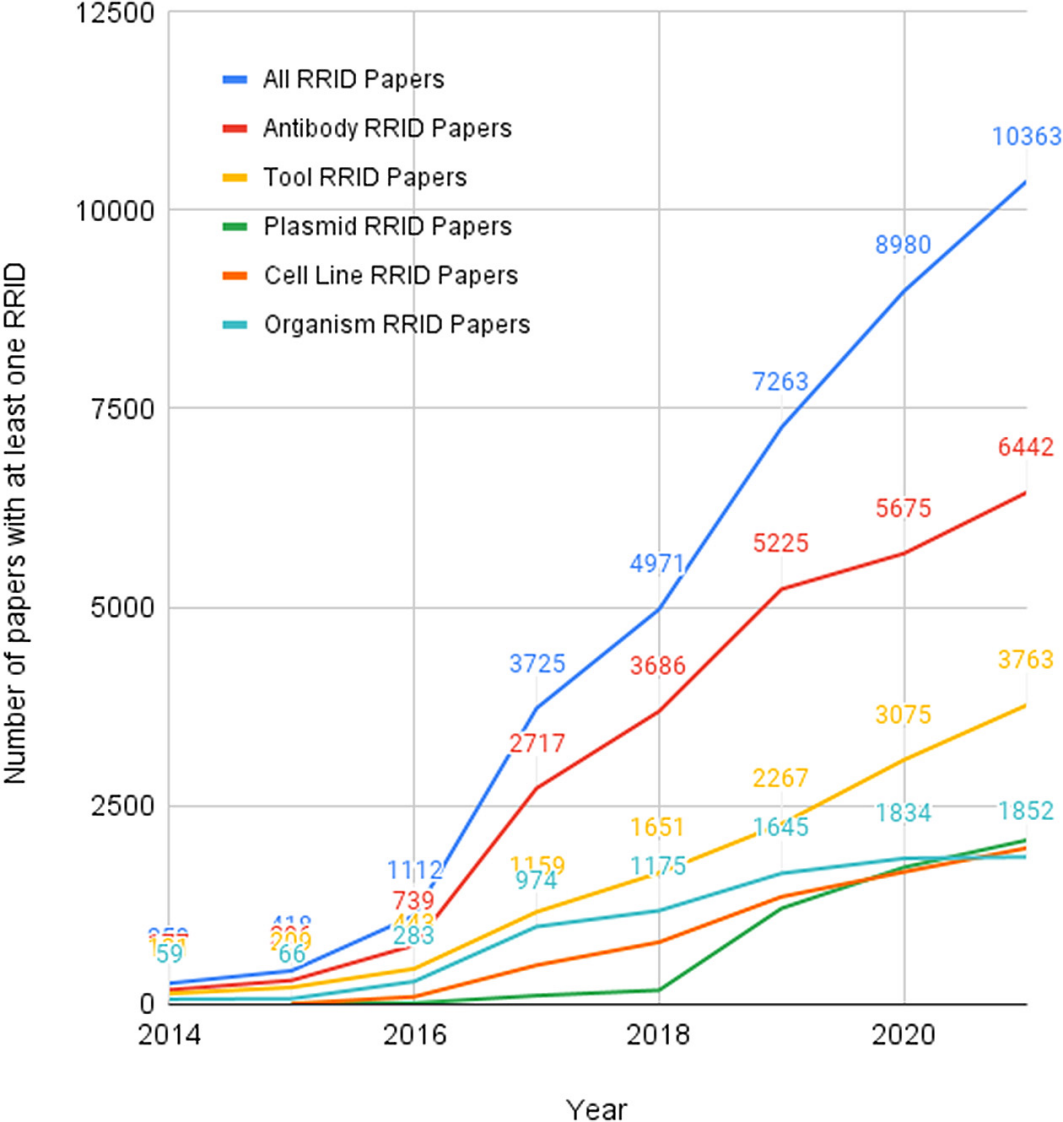
Number of papers per year.

RRIDs.org is a California nonprofit organization; the main asset being the ownership of the RRIDs themselves - keeping them free to reuse by anyone (academic, non-profit, or commercial) who wishes to improve their journal or the scientific literature. The information about the use of reagents, that a particular paper used a particular resource is delivered daily to the public Hypothes.is group (https://hypothes.is/users/SciBot) and is being made available via the CrossRef Event database (https://www.crossref.org/categories/event-data/). While there is a long path to full adoption of RRIDs in the scientific literature, the number of papers and the number RRIDs is rising rapidly demonstrating that authors can add these to their manuscripts (Table 1 and Fig. 1). This is leading to scientific literature that is more transparent and more reproducible, at least as far as the ability to repeat the experiment is concerned -  an important step. RRIDs are also beginning to shed light on a previously dark section of a manuscript -  the reagents and methods.

**Table 1:** RRIDs per resource type.

	Number of RRIDs	Number of RRID Papers	Number RRIDs per paper
Antibodies	305524	26352	11.59
Tools	53773	13460	4.00
Plasmids	13540	5764	2.35
Cell Lines	18465	6848	2.70
Organisms	29510	8345	3.54

## List of abbreviations

ABRF: Association of Biomolecular Resource Facilities; MOD: model organism databases; RRID: Research resource identifier; STAR: Structured, Transparent, Accessible Reporting

## Data availability

RRID citation data used in this report can be obtained in several ways:

1. via SciCrunch. Each RRID is associated with the currently detected RRIDs, example https://scicrunch.org/resolver/RRID:SCR_003070 (push the “view full usage report” button to view or download the data, .json or .xml extensions allow developers to access data)
2. via the Hypothes.is front end and well documented API; this is updated daily;  https://hypothes.is/users/SciBot
3. via CrossRef's event database, example https://www.crossref.org/services/event-data/
4. data from 2021 can also be obtained via the twitter https://twitter.com/robotrrid

## Competing interests

AB is a co-founder and CEO of SciCrunch Inc.

## Funding

We would like to acknowledge the following grants for funding part of this work: R43 OD024432/OD/NIH HHS/United States, R44 MH119094/MH/NIMH NIH HHS/United States; U24 DA039832/DA/NIDA NIH HHS/United States; U24 DK097771/DK/NIDDK NIH HHS/United States.

## Authors' information

Dr. Bandrowski was a founding member of the original RRID initiative and currently runs the project. She is a biologist by training but has been working with data for most of her career. As of 2016, Dr. Bandrowski is co-founder and president of the board of SciCrunch Inc, a company that is devoted to improving the scientific literature.

## Editor's Note

This commentary is part of a series to celebrate a Decade of GigaScience, to coincide with the 10th anniversary of our launch in July 2012. These papers take a look back at 10 years of advances in large-scale research as open science has become mainstream.

The first RRID integrated into a *GigaScience* manuscript was for a cell-line in April 2017, but the use really took off with the registration of software tools, and more recently scientific instruments like sequencers, which are much more of an area of focus for the journal. This has initially been a manual process by the Editors, but submitters of software are now asked to register their new tools, and it has been encouraging seeing more and more authors adding these PIDs into the papers themselves to make our papers more reproducible and usable by our human and machine readers.
